# Dual Bioconversion Strategy: Synergistic Germination and *Lactobacillus* Fermentation Engineering for a γ-Aminobutyric Acid-Enriched Beverage from Brown Rice

**DOI:** 10.3390/foods14152733

**Published:** 2025-08-05

**Authors:** Di Yuan, Shan Zhang, Bin Hong, Shan Shan, Jingyi Zhang, Dixin Sha, Shiwei Gao, Qing Liu, Shuwen Lu, Chuanying Ren

**Affiliations:** 1 Food Processing Research Institute, Heilongjiang Academy of Agricultural Sciences, Harbin 150086, China; yuandi199707@163.com (D.Y.); zhangshanfood@163.com (S.Z.); gru.hb@163.com (B.H.); 18845896856@163.com (S.S.); 18846080235@139.com (J.Z.); shadixin1997@163.com (D.S.); 2 Heilongjiang Province Key Laboratory of Food Processing, Harbin 150086, China; 3 Heilongjiang Province Engineering Research Center of Whole Grain Nutritious Food, Harbin 150086, China; 4 Suihua Branch of Heilongjiang Academy Agricultural Sciences, Suihua 152001, China; gaoshiwei1118@126.com (S.G.); liuqing58627@163.com (Q.L.)

**Keywords:** γ-aminobutyric acid (GABA), germination, *Lactobacillus* fermentation, germinated brown rice (GBR), beverage

## Abstract

Growing demand for plant-based nutraceuticals drives the need for innovative bioprocessing strategies. This study developed an integrated approach combining germination and Lactobacillus-mediated fermentation to produce a γ-aminobutyric acid (GABA)-enriched functional beverage from brown rice. Systematic screening identified an optimal rice cultivar for germination. Sequential enzymatic liquefaction and saccharification were optimized to generate a suitable hydrolysate. Screening of 13 probiotic strains revealed that a 10-strain *Lactobacillus–Bifidobacterium* consortium maximized GABA synthesis (12.2 mg/100 g). Fermentation parameters were optimized to 0.25% monosodium glutamate, 4% inoculum, 10 μmol/L pyridoxine hydrochloride, 37 °C, and 24 h. The resulting beverage achieved significantly elevated GABA concentrations while exhibiting low fat (0.2 g/100 g), reduced caloric content (233.6 kJ/100 g), and high viable probiotic counts (2 × 10^8^ CFU/g). This strategy demonstrates significant potential for the scalable production of multifunctional, plant-based nutraceuticals with targeted bioactive components.

## 1. Introduction

Gamma-aminobutyric acid (GABA), a non-protein amino acid, plays a critical role as an inhibitory neurotransmitter in the central nervous system, contributing to stress relief, blood pressure regulation, and potential therapeutic effects against chronic diseases such as diabetes and obesity [1,2]. Despite its health benefits, dietary GABA intake remains insufficient due to limited natural sources and the predominance of synthetic supplements, which often raise consumer concerns over safety and bioavailability. Although GABA-enriched functional foods and beverages have emerged, their diversity and efficacy are constrained by low GABA yields and reliance on non-dairy matrices unsuitable for lactose-intolerant or vegan consumers [3,4]. Thus, developing plant-based, naturally fermented GABA-rich beverages represents a promising avenue to address these gaps. However, inherent challenges such as potential sensory alterations and the need for specialized processing to maintain GABA stability during storage must be considered for product acceptance and functionality.

Brown rice, a nutrient-dense whole grain, serves as an excellent substrate for GABA biosynthesis due to its inherent GABA content in bran layers [5]. Germination further amplifies GABA levels by activating endogenous enzymatic pathways, yet commercial-scale production of germinated brown rice faces economic and technical challenges, including prolonged processing times and inconsistent quality [6,7]. To overcome these limitations, synergistic strategies combining germination with microbial fermentation have gained attention [8]. Lactic acid bacteria (LAB), particularly GABA-producing strains, offer a sustainable solution by converting glutamic acid into GABA during fermentation, enhancing both yield and functionality [9,10]. Beyond GABA biosynthesis, LAB consortia play multifaceted roles in plant-based beverage engineering. They critically modulate sensory profiles by metabolizing bitter compounds and generating flavor-enhancing volatiles (e.g., alcohols and esters) while simultaneously improving texture and stability [11]. Furthermore, regular consumption of LAB-fermented beverages confers functional benefits analogous to dairy probiotics—including gut microbiota modulation, immune enhancement, and mitigation of diseases of civilization like hypertension and metabolic disorders [12,13]. However, the selection of robust LAB strains and optimization of bioconversion conditions remain critical to maximizing GABA production and holistic functionality in plant-based matrices.

We hypothesized that integrating germination (to activate endogenous GABA pathways) with tailored probiotic fermentation would synergistically enhance GABA yield while improving beverage functionality beyond single-process approaches. The integration of germination and LAB fermentation—termed a “dual bioconversion” approach—holds potential to synergistically enhance GABA levels while improving product palatability and nutritional value. Previous studies highlight the variability in GABA production across LAB species, necessitating systematic screening of probiotic strains [14,15]. Furthermore, optimizing enzymatic pretreatment steps, such as liquefaction and saccharification, is essential to liberate fermentable sugars and bioactive compounds, thereby fostering microbial activity and GABA accumulation [16]. Despite advancements, a comprehensive framework for engineering high-GABA beverages through this dual strategy remains underexplored, particularly in non-dairy systems. This study establishes scalable protocol bridging germination science with fermentation engineering to address this gap.

This study aimed to develop a novel GABA-enriched beverage from brown rice through a dual bioconversion strategy involving synergistic germination and *Lactobacillus* fermentation. High-GABA brown rice varieties were screened and germinated to amplify initial GABA content. Commercial yogurt starters were evaluated for GABA-producing capabilities, followed by systematic optimization of liquefaction, saccharification, and fermentation conditions to maximize yield. The final product was evaluated for GABA concentration, probiotic viability, energy, carbohydrates, and total dietary fiber. The methodological novelty resides in the integrated optimization of the entire bioprocess, from crop cultivar selection through fermentation, coupled with a 10-strain probiotic consortium to enhance GABA bioconversion efficiency, and complemented by a precise cofactor modulation strategy utilizing pyridoxine hydrochloride (vitamin B6) for cofactor optimization. By bridging germination science with fermentation engineering, this work advances the design of functional plant-based beverages, offering a scalable solution for industrial production of low-calorie, probiotic-rich beverages suitable for lactose-intolerant consumers, with immediate applications in functional food markets targeting stress-reduction and gut health.

## 2. Materials and Methods

### 2.1. Materials and Chemicals

Twenty-four brown rice varieties were sourced from nine provinces in China, including Heilongjiang, Jilin, Liaoning, Zhejiang, Sichuan, Hunan, Hubei, Anhui, and Ningxia. These comprised 16 japonica (short-grain), 7 indica (long-grain), and 1 glutinous rice variety (Table S1). Thirteen commercial yogurt starter cultures (freeze-dried powders) (Table 1) were procured from Angel Yeast Co., Ltd. (Yichang, China), Beijing Chuanxiu Technology Co., Ltd. (Beijing, China), Biosky Biotechnology Co., Ltd. (Foshan, China), and Shunde Shangchuan Biotechnology Co., Ltd. (Shunde, China). Sodium glutamate (≥99.0%, Meihua Holdings Group Co., Ltd., Langfang, Hebei, China), pyridoxine hydrochloride (vitamin B6, Macklin Biochemical Co., Ltd., Shanghai, China), acetonitrile (HPLC grade, ≥99.9%, Macklin Biochemical Co., Ltd., Shanghai, China), sodium bicarbonate (≥99.5%, Sinopharm Chemical Reagent Co., Ltd., Shanghai, China), and sodium acetate trihydrate (≥99.0%, Aladdin Biochemical Technology Co., Ltd., Shanghai, China) were used in analytical procedures. 4-dimethylaminoazobenzene-4’-sulfonyl chloride (DABS-Cl) and 3,5-dinitrosalicylic acid (DNS) were purchased from Aladdin Biochemical Technology Co., Ltd. (Shanghai, China).

### 2.2. Preparation of Germinated Brown Rice

Using brown rice (obtained after hulling paddy rice by dehusking) as the raw material, the rice was immersed in distilled water at a 1:3 (*w*/*v*) rice-to-water ratio. Following a soaking period of 4 h, the rice was sprouted through spraying. The sprouting process was conducted at a constant temperature of 30 °C for 40 h. During sprouting, grains were sprayed with temperature-equilibrated distilled water every 2 h, maintaining a consistent moisture addition of 20 mL per kg rice per spray cycle to ensure surface saturation without waterlogging. This protocol was optimized based on established methods for GABA accumulation in germinated cereals, where 30–35 °C and 36–48 h germination maximized glutamate decarboxylase activity and GABA synthesis [17,18].

### 2.3. Preparation of GBR Enzymatic Hydrolysate

According to the method described in Reference [18], with some modifications, germinated brown rice was steamed at 121 °C for 20 min and homogenized with distilled water (1:2 *w*/*v*) using an FJ-200 high-speed homogenizer (Shanghai Specimen Model Factory, Shanghai, China) to obtain a uniform slurry. The slurry underwent liquefaction through the addition of α-amylase (30 U/g) at 80 °C for 40 min, followed by saccharification with glucoamylase (75 U/g) at 60 °C for 1 h. Enzymatic reactions were terminated by boiling the hydrolysate for 15 min. The resulting hydrolysate was stored at 4 °C until subsequent utilization.

### 2.4. Determination of Centrifugal Sedimentation Rate

The centrifugal sedimentation rate was determined following the method described in previous reports [19]. A 10 mL aliquot of the liquefied hydrolysate was transferred into a graduated centrifuge tube and centrifuged at 4000 rpm for 20 min. After discarding the supernatant, the tube was inverted for 5 min to drain the residual liquid. The mass of the sediment was measured and expressed as a percentage of the initial hydrolysate mass using the following formula:

(1) Sedimentation rate (%) = Mass of Sediment (g)/Mass of Hydrolysate (g) × 100

### 2.5. Determination of Dextrose Equivalent (DE)

Reducing Sugar Content: The reducing sugar content was quantified using the 3,5-dinitrosalicylic acid (DNS) method [20]. Following centrifugation of 5 g enzymatic hydrolysate at 3500 rpm for 10 min, the supernatant was collected. Specifically, 1.0 mL of appropriately diluted sample supernatant was mixed with 1.0 mL DNS reagent in a test tube. The mixture was vortexed, heated in a boiling water bath for exactly 5 min to develop the red-brown color, then immediately cooled in an ice-water bath for 10 min. After adding 8 mL distilled water, absorbance was measured at 540 nm using a spectrophotometer (UV-1800, Shimadzu, Japan). A standard glucose curve was used to calculate the reducing sugar concentration.

Soluble Solids Content: For soluble solids determination, 10 g of the hydrolysate was centrifuged at 3000 rpm for 20 min at 4 °C. The supernatant was filtered through a 0.45 μm membrane filter, and the soluble solids content was measured using an LB90T refractometer (Guangzhou Suwei Electronic Technology Co., Ltd., Guangzhou, China).

DE Value Calculation: The dextrose equivalent (DE) was calculated using the following formula:

(2) DE(%) = Reducing Sugar Content (g/100 g)/Soluble Solids Content (g/100 g) × 100

### 2.6. Screening of Fermentation Strains

The GBR hydrolysate was supplemented with 0.25% (*w*/*v*) monosodium glutamate (MSG) to enhance GABA biosynthesis [21]. To enhance GABA biosynthesis, pyridoxine hydrochloride was added to the GBR hydrolysate at concentrations ranging from 5 to 50 μmol/L prior to fermentation. This cofactor optimization was evaluated in conjunction with the selected probiotic consortium. Each of the 13 starter cultures (5% *w*/*v* inoculum) was added to the hydrolysate and fermented at 30 °C for 24 h. Fermentation was conducted in GHP-9080 temperature-controlled incubators (Shanghai Jinghong Laboratory Instruments, Shanghai, China). Samples (1.5 mL) were collected, centrifuged (10,000 r/min, 10 min, 4 °C), and filtered through a 0.22 μm membrane. The supernatant was analyzed for GABA content using high-performance liquid chromatography (HPLC).

### 2.7. Optimization Experimental Design

Systematic optimization of critical parameters was conducted through controlled one-variable-at-a-time experiments. For slurry viscosity assessment, germinated brown rice was homogenized with distilled water at ratios of 1:0.5, 1:1, 1:2, 1:4, 1:6, and 1:8 (*w*/*v*) using an homogenizer. Liquefaction parameters were optimized by varying α-amylase dosage (10–50 U/g), incubation time (20–60 min), and temperature (50–90 °C), with the centrifugal sedimentation rate as the evaluation metric. Saccharification conditions were tested across glucoamylase concentrations (25–150 U/g), duration (0.5–4 h), and temperatures (50–70 °C), measuring the dextrose equivalent. Fermentation variables included monosodium glutamate supplementation (0–0.2% *w*/*v*), inoculum size (0–8% *w*/*v*), pyridoxine hydrochloride concentration (0–100 μmol/L), and fermentation period (0–72 h at 37 °C), with GABA quantification by HPLC after each trial.

### 2.8. Determination of GABA Content by HPLC

The determination of GABA content was performed in accordance with NY/T 2890-2016 [22]. An aliquot of 1.0 mL supernatant was accurately transferred and mixed with 0.20 mL of a 4% (*w*/*v*) aqueous sodium bicarbonate solution and 0.40 mL of a 0.2% (*w*/*v*) aqueous solution of 4-dimethylaminoazobenzene-4’-sulfonyl chloride (DABS-Cl). The mixture was subjected to a derivatization reaction at 70 °C for 20 min in a water bath. The resulting solution was filtered through a 0.22 μm microporous membrane prior to chromatographic analysis.

Chromatographic analysis was performed using a Thermo Scientific UltiMate 3000 system equipped with an online degasser, a binary pump, and a UV detector. Separation was achieved using a C18 column (4.6 mm × 250 mm, 5 µm particle size) maintained at 30 °C. Detection was carried out at a wavelength of 436 nm. The mobile phase consisted of acetonitrile (eluent A) and an aqueous solution of sodium acetate trihydrate (6.8 g/L) (eluent B). Chromatographic separation employed an isocratic elution program with 35% eluent A. The total run time was 15 min at a flow rate of 1.0 mL/min, with an injection volume of 10 µL.

The developed HPLC method was validated for the quantification of GABA in terms of linearity, sensitivity, and precision. Linearity was assessed by analyzing a series of standard GABA solutions ranging from 1 μg/mL to 50 μg/mL. The method exhibited good linearity over this range, with a correlation coefficient (R^2^) of 0.9992. The limit of detection (LOD) and limit of quantification (LOQ) were determined based on signal-to-noise ratios of 3:1 and 10:1, respectively, yielding values of 0.3 μg/mL and 1 μg/mL. Method precision was evaluated as intra-day (repeatability) and inter-day variability by analyzing three replicates of a standard GABA solution at 5 μg/mL on the same day and over three consecutive days, respectively. The relative standard deviations (RSD) for intra-day and inter-day precision were <3.8% and <5.9%, respectively, indicating acceptable precision.

### 2.9. Nutrient Composition Analysis

The determination of key nutritional components in the samples was strictly performed according to the National Standards of the People’s Republic of China currently in effect. The specific analytical methodologies employed were as follows. Energy: Calculated indirectly using the energy conversion factors specified in GB/Z 21922-2008, i.e., 17 kJ/g for protein, 37 kJ/g for fat, and 17 kJ/g for carbohydrates [23]. Fat: Quantitatively calculated via the Soxhlet extraction system (B-811, Büchi Labortechnik AG, Flawil, Switzerland) (Method I), as prescribed in National Food Safety Standard—GB 5009.6-2016, using anhydrous ethyl ether as the extraction solvent [24]. Protein: Determined using the Kjeldahl method referenced in National Food Safety Standard—GB 5009.5-2016 [25], performed with an automatic nitrogen analyzer (Kjeltec 8400, FOSS Analytical, Hillerød, Denmark). Total protein content was calculated using a nitrogen-to-protein conversion factor of 6.25 [25]. Carbohydrates: Calculated by the difference method according to the “Carbohydrate” calculation principles outlined in GB/Z 21922-2008 (i.e., 100% minus the sum of the percentages of moisture, ash, protein, and fat) [23]. Lactic Acid Bacteria Count: Enumerated following the methodology stipulated in National Food Safety Standard—GB 4789.35-2023: Lactic Acid Bacteria Examination [26]. Samples were homogenized in sterile saline, serially diluted, plated on MRS agar, and incubated under anaerobic conditions at 36 ± 1 °C for 48 ± 2 h. Results are expressed as colony-forming units per gram (CFU/g). Sodium: Determined using flame atomic absorption spectrometry (Method 3), as specified in National Food Safety Standard—GB 5009.268-2016, using deuterium lamp background correction and matrix-matched calibration standards [27]. Total Dietary Fiber: Quantified by enzymatic-gravimetric method, as referenced in GB 5009.88-2023 [28]. Reducing Sugars: Determined by DNS method as described in Section 2.5. GABA: Analyzed by HPLC with DABS-Cl derivatization as detailed in Section 2.8. All determinations were conducted using triplicate independent samples. Strict quality control measures were implemented throughout the analytical procedures, including blank controls, calibration with certified reference materials, and spike recovery tests, to ensure data accuracy and reproducibility.

### 2.10. Statistical Analysis

All of the samples were analyzed in triplicate, and the data were calculated as the average ± standard deviation (SD). Statistical analysis was performed using the SPSS statistics 22 (SPSS Inc., Chicago, IL, USA). Significant differences were determined by one-way analysis of variance (ANOVA), followed by Duncan’s multiple range test at *p* < 0.05.

## 3. Results and Discussion

### 3.1. Screening of High-GABA Brown Rice Varieties

Significant variation in GABA content was observed among cultivars in both raw and germinated brown rice (*p* < 0.05, Table 2), necessitating systematic selection of varieties suitable for high-GABA fermented beverage production. Twenty-four newly harvested rice varieties from nine Chinese provinces were germinated under controlled conditions (4 h soaking at room temperature, followed by 40 h germination at 30 °C, with intermittent rinsing). Quantification revealed distinct genotypic differences in GABA accumulation patterns, consistent with established literature highlighting genetic regulation of GABA synthesis pathways [29,30].

Post-germination GABA content differed significantly across cultivars (Table 2). Suijing 309 demonstrated the highest mean content (29.68 ± 0.12 mg/100 g; group “a”), forming a statistically distinct group. This was significantly superior to cultivars in subsequent statistical groups, including Suijing 18 (28.63 ± 0.08 mg/100 g; group “b”), which clustered with Ningjing 48 (25.92 ± 0.28 mg/100 g) and Xinjing 64 (24.06 ± 0.17 mg/100 g). Based on its significantly elevated post-germination GABA accumulation, combined with favorable agronomic and culinary properties, Suijing 309 was selected for subsequent fermentation studies.

### 3.2. Optimization of Slurry Water-to-Rice Ratio

The texture of germinated brown rice slurry was evaluated at varying water-to-rice ratios (Table 3). A 1:2 ratio yielded a viscous yet stirrable consistency, ideal for enzymatic hydrolysis and fermentation.

### 3.3. Optimization of Liquefaction Conditions

Liquefaction is a critical step in the production of germinated brown rice beverages, as it hydrolyzes starch into smaller saccharides (e.g., maltose, maltotriose, and maltooligosaccharides) while partially degrading proteins and cellulose. This process prevents starch retrogradation, enhances beverage clarity, and improves product stability by reducing sedimentation [31]. The liquefied hydrolysate serves as an ideal substrate for probiotic growth, enabling the development of fermented beverages with high viable cell counts. Thermostable α-amylase, which randomly cleaves α-1,4 glycosidic bonds within starch molecules, was employed for liquefaction. The efficiency of this enzymatic reaction depends on enzyme dosage and activity. Centrifugal sedimentation rate (CSR), inversely correlated with liquefaction efficiency, was used as the primary evaluation metric.

#### 3.3.1. Enzyme Dosage Optimization

**Figure 1:**
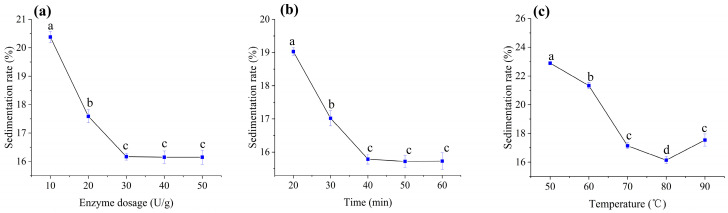
Effects of liquefaction parameters on centrifugal sedimentation rate: (**a**) enzyme dosage, (**b**) time, and (**c**) temperature. Mean values with different letters (a, b, c, d) differ significantly (*p* < 0.05) by Duncan’s multiple range test. The letter ’a’ represents the group with the highest mean, followed by b, c, and d in decreasing order.

Under fixed conditions (80 °C, 30 min), CSR decreased progressively as α-amylase dosage increased from 10 to 30 U/g (Figure 1a). At 30 U/g, CSR reached a minimum of 16.17%, beyond which no reduction was observed. Unchanged sedimentation rates above 30 U/g suggest saturation of starch granule binding sites, with unhydrolyzed starch retrograding into resistant structures. Thus, 30 U/g was selected as the optimal enzyme dosage.

#### 3.3.2. Time Optimization

Using 30 U/g α-amylase at 80 °C decreased CSR sharply within the first 40 min (Figure 1b), indicating rapid starch hydrolysis. Prolonging liquefaction beyond 40 min yielded negligible improvements, likely due to the exhaustion of accessible starch substrates. To balance efficiency and industrial feasibility, 40 min was identified as the optimal duration.

#### 3.3.3. Temperature Optimization

Temperature critically influences enzyme activity. At 30 U/g α-amylase and 30 min, CSR decreased with temperatures increasing from 50 °C to 80 °C (Figure 1c), reflecting enhanced enzyme kinetics. However, at 90 °C, CSR increased significantly, attributed to partial enzyme denaturation and reduced catalytic efficiency. These findings align with the thermostability profile of α-amylase, which exhibits peak activity near 80 °C but degrades rapidly at higher temperatures [32]. Consequently, 80 °C was chosen as the optimal liquefaction temperature.

### 3.4. Optimization of Saccharification Conditions

Glucoamylase catalyzes the hydrolysis of α-1,6 glycosidic bonds in dextrins, which remain unhydrolyzed after α-amylase treatment to produce glucose [33]. To maximize starch conversion, saccharification was performed on liquefied germinated brown rice hydrolysate, with dextrose equivalent (DE) serving as the primary evaluation metric.

#### 3.4.1. Enzyme Dosage Optimization

**Figure 2:**
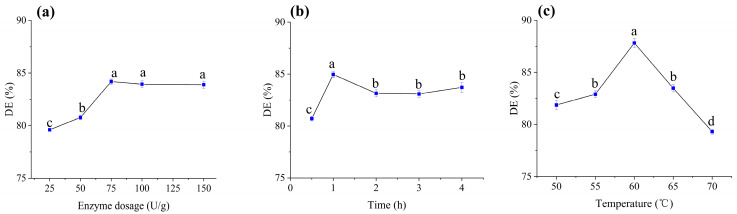
Effects of saccharification parameters on DE value: (**a**) enzyme dosage, (**b**) time, and (**c**) temperature. Mean values with different letters (a, b, c, d) differ significantly (*p* < 0.05) by Duncan’s multiple range test. The letter ’a’ represents the group with the highest mean, followed by b, c, and d in decreasing order.

Under fixed conditions (65 °C, 2 h), DE increased proportionally with glucoamylase dosage from 25 to 75 U/g, peaking at 84.19% (Figure 2a). Further increases in enzyme dosage (up to 150 U/g) yielded no significant improvement, indicating substrate saturation. Thus, 75 U/g was selected as the optimal enzyme dosage.

#### 3.4.2. Time Optimization

Using 75 U/g glucoamylase at 65 °C, DE rose sharply within the first hour (Figure 2b), reflecting rapid hydrolysis of accessible glycosidic bonds. Prolonging saccharification beyond 1 h resulted in a gradual decline and stabilization of DE, likely due to enzyme inactivation or depletion of hydrolysable substrates. To balance efficiency and energy consumption, 1 h was identified as the optimal duration.

#### 3.4.3. Temperature Optimization

Enzyme activity is highly temperature-dependent. At 75 U/g and 2 h, DE increased with temperature up to 60 °C, reaching a maximum of 87.82% (Figure 2c). Above 60 °C, DE declined sharply, attributed to thermal denaturation of glucoamylase. These results align with the thermostability profile of glucoamylase, which exhibits peak activity near 60 °C but rapidly loses structural integrity at higher temperatures [34]. Consequently, 60 °C was chosen as the optimal saccharification temperature.

### 3.5. Optimization of Fermentation Conditions

#### 3.5.1. Screening of Fermentation Strains

**Figure 3:**
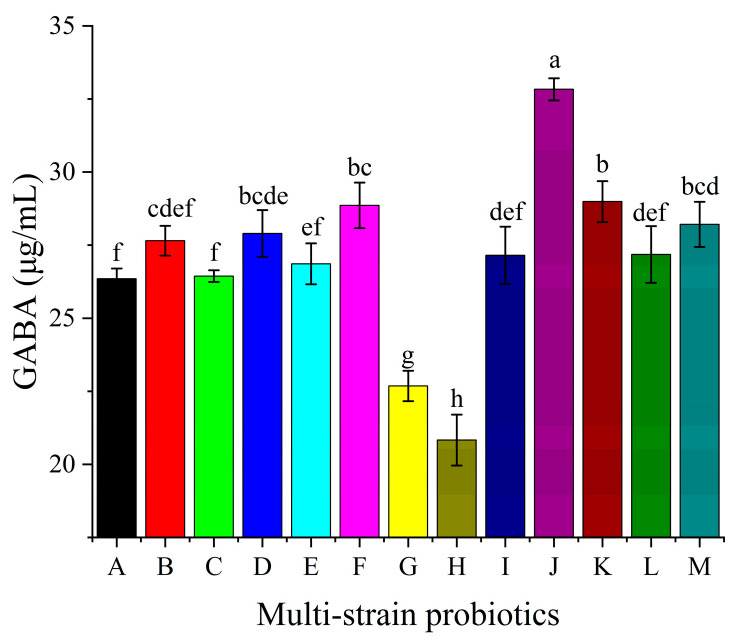
GABA production by different multi-strain probiotics (A–M). Mean values with different letters differ significantly (*p* < 0.05) by Duncan’s multiple range test. The letter ’a’ represents the group with the highest mean, followed by b, c, d, e, f,g and h in decreasing order.

Recent studies have identified several probiotic lactic acid bacteria (LAB) strains, including *Lactococcus lactis*, *Leuconostoc mesenteroides*, *Streptococcus thermophilus*, and *Lactobacillus plantarum*, as efficient producers of GABA via microbial biosynthesis [35]. To identify the most effective starter culture, 13 commercial multi-strain probiotics (labeled A–M) were evaluated for their GABA-producing capabilities in germinated brown rice hydrolysate. As shown in Figure 3, culture J yielded the highest GABA content, exceeding other starters by 1.13- to 1.58-fold. Culture J comprises a 10-strain consortium, including *Lactobacillus delbrueckii* subsp. *bulgaricus, Streptococcus thermophilus, Lactococcus lactis* subsp. *lactis, Lactococcus lactis* subsp. *cremoris, Lactococcus lactis* subsp. *lactis* biovar *diacetylactis, Leuconostoc mesenteroides* subsp. *mesenteroides, Lactobacillus acidophilus, Bifidobacterium animalis* subsp. *lactis, Bifidobacterium longum,* and *Bifidobacterium infantis*.

This consortium encompasses multiple GABA-producing species, suggesting synergistic interactions that enhance substrate utilization and GABA synthesis. Consequently, culture J was selected for subsequent experiments.

#### 3.5.2. Effect of Monosodium Glutamate (MSG) Supplementation

**Figure 4:**
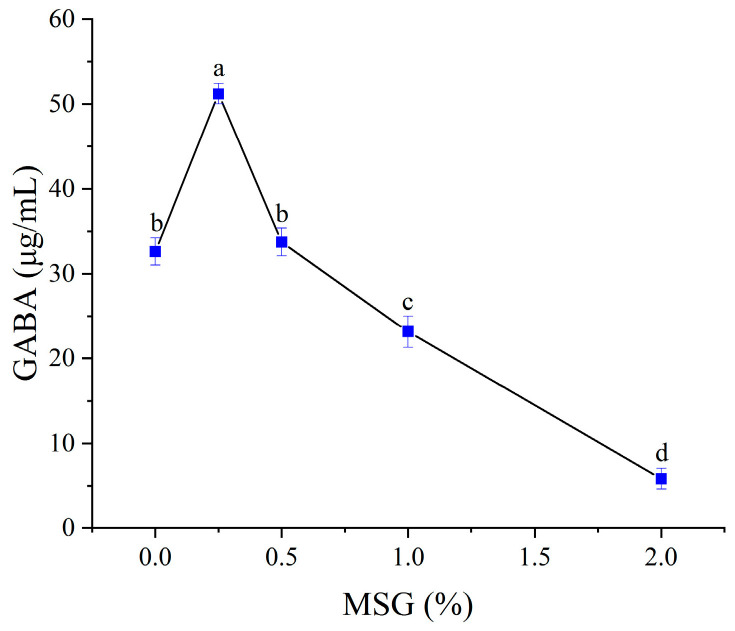
Effect of MSG on GABA content. Mean values with different letters (a, b, c, d) differ significantly (*p* < 0.05) by Duncan’s multiple range test. The letter ’a’ represents the group with the highest mean, followed by b, c, and d in decreasing order.

Glutamate decarboxylase (GAD), a pyridoxal 5’-phosphate (PLP)-dependent enzyme, catalyzes the irreversible decarboxylation of L-glutamate to GABA. Since most LAB strains cannot synthesize sufficient endogenous L-glutamate, exogenous supplementation with MSG—a precursor hydrolyzed to L-glutamate—is critical for maximizing GABA yield [36]. As illustrated in Figure 4, GABA content increased proportionally with MSG addition up to 0.25% (*w*/*v*), beyond which it declined. This biphasic trend aligns with prior reports: moderate MSG levels enhance GAD activity by upregulating amino acid metabolism, while excessive MSG inhibits microbial growth via osmotic stress or negatively regulates GAD expression [37]. Thus, 0.25% MSG was identified as the optimal supplementation level.

#### 3.5.3. Effect of Probiotic Inoculum Size

**Figure 5:**
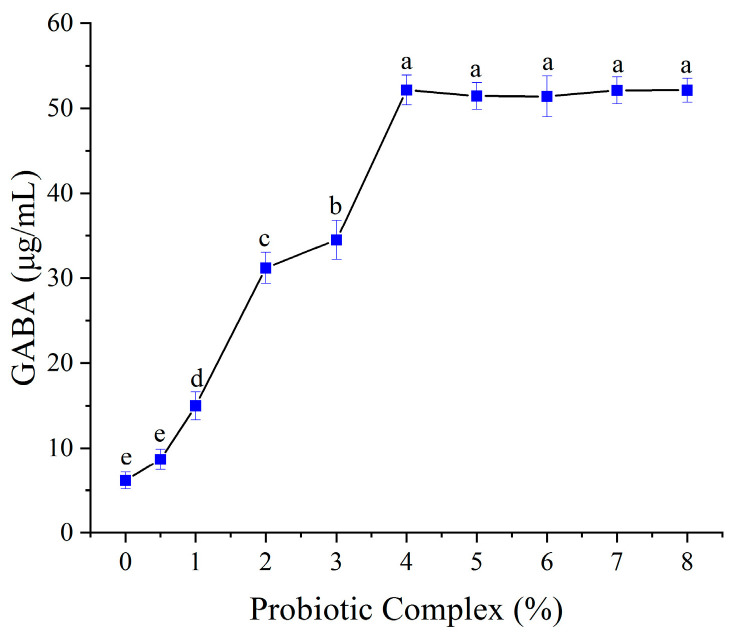
Effect of probiotic inoculum size on GABA content. Mean values with different letters differ significantly (*p* < 0.05) by Duncan’s multiple range test. The letter ’a’ represents the group with the highest mean, followed by b, c, d and e in decreasing order.

The relationship between probiotic inoculum size and GABA production is shown in Figure 5. GABA content increased linearly with inoculum size up to 4% (*w*/*v*), after which it plateaued. This suggests that higher inoculum densities initially promote the breakdown of complex substrates (e.g., proteins and polysaccharides) into bioavailable amino acids, including L-glutamate, thereby driving GABA synthesis [38]. However, substrate limitation likely restricts further GABA accumulation beyond 4% inoculum. Consequently, 4% was selected as the optimal inoculum size.

#### 3.5.4. Fermentation Time and pH Dynamics

**Figure 6:**
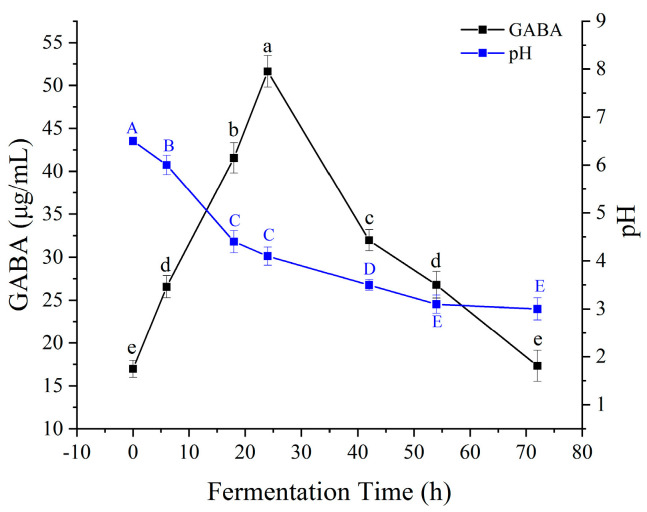
Temporal profiles of GABA content and pH during fermentation. Uppercase letters (A–E) and lowercase letters (a–e) indicate significant differences (*p* < 0.05) among means within their respective data groups by Duncan’s multiple range test. For each letter system, the letter ’A’ (or ’a’) denotes the highest mean value, followed by subsequent letters (B, C, D, E / b, c, d, e) in decreasing order. Letters are not comparable between the two systems.

GABA synthesis and pH changes during fermentation are depicted in Figure 6. GABA content peaked at 24 h, followed by a gradual decline, while pH decreased continuously from 6.2 to 3.8. The initial rise in GABA correlates with lactic acid accumulation, which lowers pH and activates acid-stress-responsive GAD enzymes [39]. The subsequent decline in GABA after 24 h may be attributed to the activation of GABA degradation pathways. Although low pH favors GAD activity, prolonged fermentation under these conditions, coupled with potential depletion of L-glutamate, might induce GABA transaminase (GABA-T) activity. GABA-T converts GABA into succinic semialdehyde, which then enters the tricarboxylic acid cycle [35]. These findings underscore the importance of controlled fermentation duration to balance synthesis and degradation.

#### 3.5.5. Pyridoxine Hydrochloride Supplementation

**Figure 7:**
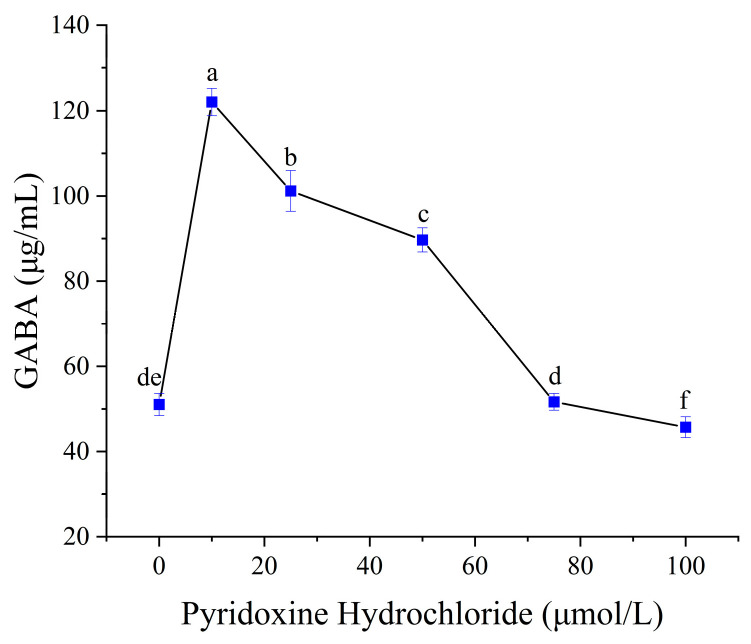
Effect of pyridoxine hydrochloride concentration on GABA synthesis. Mean values with different letters differ significantly (*p* < 0.05) by Duncan’s multiple range test. The letter ’a’ represents the group with the highest mean, followed by b, c, d, e and f in decreasing order.

Pyridoxine hydrochloride, a precursor of the GAD cofactor PLP, was tested as a cost-effective alternative to commercial PLP. As shown in Figure 7, GABA content increased with pyridoxine supplementation up to 10 μmol/L, beyond which no further enhancement occurred. This plateau contrasts with studies reporting continuous GABA increases at higher pyridoxine levels, likely due to strain-specific PLP biosynthesis capacities or differences in media composition [40]. These results highlight the necessity of strain- and matrix-specific optimization for cofactor strategies.

### 3.6. Final Beverage Production Protocol

The GABA-enriched brown rice beverage was produced through a dual bioconversion strategy integrating synergistic germination and multi-strain fermentation. As shown in Table 4, the high-GABA cultivar Suijing 309 underwent controlled germination involving a 4 h soaking at ambient temperature, followed by a 40 h sprouting at 30 °C, with bi-hourly hydration. The germinated grains were homogenized with distilled water at a 1:2 (*w*/*v*) ratio to achieve optimal slurry viscosity, then sequentially hydrolyzed via thermostable α-amylase (30 U/g, 80 °C, 40 min) and glucoamylase (75 U/g, 60 °C, 1 h) to generate fermentable hydrolysate. Fermentation employed a defined 10-strain probiotic consortium (*Lactobacillus delbrueckii* subsp. *bulgaricus*, *Streptococcus thermophilus*, *Lactococcus lactis* subsp. *lactis*, *Lactococcus lactis* subsp. *cremoris*, *Lactococcus lactis* subsp. *lactis* biovar *diacetylactis*, *Leuconostoc mesenteroides* subsp. *mesenteroides*, *Lactobacillus acidophilus*, *Bifidobacterium animalis* subsp. *lactis*, *Bifidobacterium longum*, *Bifidobacterium infantis*) inoculated at 4% (*w*/*v*) into MSG-supplemented hydrolysate (0.25% *w*/*v*). The bioconversion was enhanced with 10 μmol/L pyridoxine hydrochloride and conducted at 37 °C for 24 h (final pH, 3.8). This integrated protocol established the foundation for nutritional characterization of the functional beverage.

### 3.7. Nutrient Value

The sequential bioconversion strategy—germination followed by fermentation—significantly enhanced γ-aminobutyric acid content within the plant-based matrix. Germination of Suijing 309 brown rice increased precursor availability, yielding a 10.8-fold GABA elevation compared to that of raw material (Table 2), while enzymatic hydrolysis liberated essential reducing sugars (14.6 g/100 g). Subsequent fermentation, driven by lactic acid bacteria, further modulated the product profile: decarboxylation elevated GABA to a final concentration of 12.2 mg/100 g (Figure 4), microbial metabolism generated desirable sensory notes such as diacetyl (attributed to *Lactococcus lactis* in Consortium J, Table 1) and mild acidity (pH 3.8), and reduced caloric density (233.6 kJ/100 g) through sugar utilization. This dual-stage approach achieved GABA levels competitive with those of advanced fermentation-enhanced cereal beverages [18], uniquely leveraging germination-derived precursors for potentially improved GABA bioavailability (via natural metabolite complexes) and enhanced stability through probiotic-mediated acidification.

The resulting germinated brown-rice fermented beverage exhibits a nutritionally optimized profile (Table 5), characterized by low fat content (0.2 g/100 g) significantly below that of conventional dairy-based probiotic drinks, aligning with reduced-fat dietary trends [41]. Its carbohydrate composition (13.45 g/100 g) primarily derives from enzymatic and fermentative liberation of reducing sugars, contributing to natural sweetness while maintaining lower energy density than that of sucrose-sweetened beverages. Sodium levels were moderate (112.2 mg/100 g), conducive to electrolyte balance, although protein content (0.057 g/100 g) lags behind that of fermented soybean beverages, suggesting future plant-protein fortification opportunities [31]. Critically, the beverage demonstrates substantial functional potential: the quantified GABA concentration (12.2 mg/100 g) falls within the clinically effective range associated with stress reduction and mild blood pressure modulation in human studies upon consumption of standard servings [1,2] and is comparable to values reported in recent studies for optimized fermented brown rice beverages [18]. Concurrently, the high viable probiotic count (2 × 10^8^ CFU/g) meets or exceeds levels commonly cited in the literature as necessary to potentially confer gastrointestinal benefits upon consumption of standard servings, supporting the product’s potential for gut health [41,42]. Maintaining these favorable sensory attributes (flavor balance, texture) and nutritional integrity during scale-up requires careful process optimization.

Despite the significant potential for GABA enrichment demonstrated by this dual bioconversion strategy, extending it to commercial production presents key challenges. The germination step necessitates precise, resource-intensive control of temperature, humidity, and aeration over an extended period (40 h). Ensuring sterility during large-scale enzymatic hydrolysis and fermentation is paramount to prevent contamination and safeguard probiotic viability. Furthermore, optimizing the cost-effectiveness of enzyme usage and pyridoxine hydrochloride supplementation, alongside managing the relatively long fermentation time (24 h) compared to that of some dairy fermentations, are crucial factors determining industrial viability [6,31]. Process integration and automation will be essential to address these challenges effectively, ensuring the translation of the laboratory-scale achievements in GABA accumulation, probiotic viability, nutritional profile, and sensory quality to a commercially feasible process.

### 3.8. Considerations for Product Stability

The stability and probiotic viability of GABA during storage is crucial for the functional efficacy of the final beverage. While this study focused on optimizing production, GABA degradation during storage in fermented plant matrices has been reported, potentially influenced by residual enzyme activity, pH shifts, microbial metabolism, or oxidation [4]. Maintaining low temperatures and potentially optimizing packaging (e.g., using an oxygen barrier) are common strategies to mitigate GABA loss and preserve probiotic counts in similar functional beverages [41,42]. Future studies should directly assess the shelf-life stability of GABA concentration and probiotic viability under recommended storage conditions for this specific product.

## 4. Conclusions

This study establishes a dual bioconversion strategy integrating synergistic germination and multi-strain *Lactobacillus*-dominated fermentation to engineer a high-value functional beverage from brown rice. By leveraging germination-induced enzymatic activation to elevate precursor (glutamate) bioavailability and subsequent co-fermentation with a rationally selected probiotic consortium, we achieve GABA enrichment (12.2 mg/100 g). The optimized fermentation parameters (0.25% monosodium glutamate, 4% composite inoculum, 10 μmol/L pyridoxine hydrochloride, 24 h/37 °C) exemplify microbial engineering, where biochemical cofactor provision and strain metabolic crosstalk converge to maximize γ-aminobutyric acid biotransformation. Critically, the dual-process design concurrently enhanced broader nutritional functionality: sustained probiotic viability and reduced energy density collectively position this beverage as a functional food addressing modern dietary needs. This work validates synergistic bio-processing as a promising paradigm for transforming staple grains into targeted nutraceutical delivery systems. The optimized parameters provide a foundation for producing GABA-enriched brown rice beverages with enhanced probiotic viability and a favorable nutritional profile. However, limitations of this study include the need for sensory optimization to ensure consumer acceptance and the evaluation of GABA and probiotic stability during product shelf-life under commercial storage conditions. Future research should focus on pilot-scale validation of the process, sensory profiling and improvement, comprehensive shelf-life assessment, and clinical evaluation of the beverage’s physiological benefits. Addressing these aspects is crucial for realizing the potential of this dual bioconversion strategy within the functional food industry.

## Figures and Tables

**Table 1:** Composite starter cultures.

Code	Product Name	Composite Starter Culture (Microbial Strains)
A	Angel Yeast Yogurt Starter (2 strains)	*Lactobacillus delbrueckii* subsp. *bulgaricus, Streptococcus thermophilus*
B	Angel Yeast Yogurt Starter (4 strains)	*Lactobacillus delbrueckii* subsp. *bulgaricus*, *Streptococcus thermophilus*, *Lactococcus lactis* subsp. *lactis*, *Bifidobacterium animalis* subsp.
C	Angel Yeast Yogurt Starter (8 strains)	*Lactobacillus delbrueckii* subsp. *bulgaricus*, *Streptococcus thermophilus*, *Lactobacillus acidophilus*, *Bifidobacterium bifidum*, *Lactobacillus plantarum*, *Lactobacillus rhamnosus*, *Lactobacillus casei*, *Lactobacillus reuteri*
D	Angel Yeast Yogurt Starter (10 strains)	*Lactobacillus delbrueckii* subsp. *bulgaricus*, *Streptococcus thermophilus*, *Lactobacillus acidophilus*, *Bifidobacterium longum*, *Bifidobacterium breve*, *Bifidobacterium animalis* subsp. *lactis*, *Bifidobacterium adolescentis*, *Bifidobacterium bifidum*, *Bifidobacterium infantis*, *Lactobacillus reuteri*
E	Angel Yeast Yogurt Starter (12 strains)	*Streptococcus thermophilus*, *Lactobacillus delbrueckii* subsp. *bulgaricus*, *Bifidobacterium longum*, *Bifidobacterium animalis* subsp. *lactis*, *Bifidobacterium breve*, *Bifidobacterium infantis*, *Bifidobacterium adolescentis*, *Lactobacillus acidophilus*, *Lactobacillus rhamnosus*, *Lactobacillus reuteri*, *Lactobacillus plantarum*, *Lactobacillus casei*
F	Chuanxiu (imported, 10 strains)	*Lactobacillus delbrueckii* subsp. *bulgaricus*, *Streptococcus thermophilus*, *Bifidobacterium animalis* subsp. *lactis*, *Lactobacillus acidophilus*, *Lactobacillus casei*, *Lactobacillus rhamnosus*, *Lactococcus lactis* subsp. *lactis*, *Lactococcus lactis* subsp. *cremoris*, *Leuconostoc mesenteroides* subsp. *mesenteroides*, *Lactococcus lactis* subsp. *lactis* biovar *diacetylactis*
G	Yogourmet Kefir Yogurt Starter	*Lactobacillus acidophilus*, *Lactococcus lactis* subsp. *lactis*, *Lactococcus lactis* subsp. *cremoris*, *Lactococcus lactis* subsp. *lactis* biovar *diacetylactis*, *Saccharomyces cerevisiae*, *Kluyveromyces lactis*
H	Biosky (Greek Style) Yogurt Starter Powder	*Lactobacillus delbrueckii* subsp. *bulgaricus*, *Streptococcus thermophilus*, *Lactobacillus acidophilus*, *Lactobacillus plantarum*, *Lactobacillus casei*
I	Biosky (Probiotic Type) (upgraded, 8 strains)	*Lactobacillus delbrueckii* subsp. *bulgaricus*, *Streptococcus thermophilus*, *Lactobacillus acidophilus*, *Lactobacillus plantarum*, *Lactobacillus casei*, *Bifidobacterium animalis* subsp. *lactis, Bifidobacterium longum, Lactobacillus rhamnosus*
J	Biosky (10 strains)	*Lactobacillus delbrueckii* subsp. *bulgaricus*, *Streptococcus thermophilus*, *Lactococcus lactis* subsp. *lactis*, *Lactococcus lactis* subsp. *cremoris*, *Lactococcus lactis* subsp. *lactis* biovar *diacetylactis*, *Leuconostoc mesenteroides* subsp. *mesenteroides*, *Lactobacillus acidophilus*, *Bifidobacterium animalis* subsp. *lactis*, *Bifidobacterium longum*, *Bifidobacterium infantis*
K	Chuanxiu Pickle/Kimchi Starter	*Lactobacillus plantarum*, *Lactobacillus acidophilus*, *Lactobacillus rhamnosus*
L	Biosky Pickle Starter Powder	*Lactobacillus plantarum*, *Lactobacillus acidophilus*, *Lactobacillus fermentum*
M	Shangchuan Pickle Starter	*Lactobacillus plantarum*, *Leuconostoc mesenteroides* subsp. *mesenteroides*, *Pediococcus pentosaceus*

**Table 2:** GABA content in raw and germinated brown rice across cultivars (mg/100 g).

Sample Name	Raw GABA	Germinated GABA
Zhongjiazao 17	2.96 ± 0.05 ^o^	5.44 ± 0.07 ^r^
Yixiangyou 2115	1.75 ± 0.02 ^q^	20.58 ± 0.11 ^f^
Deyou 8	2.99 ± 0.07 ^o^	19.11 ± 0.18 ^g^
Fengliangyou 4	3.74 ± 0.03 ^l^	15.48 ± 0.22 ^j^
Tiejing 11	3.55 ± 0.02 ^m^	18.19 ± 0.14 ^i^
Tonghe 899	6.83 ± 0.04 ^f^	9.09 ± 0.16 ^p^
Tongke 59	7.97 ± 0.08 ^e^	14.24 ± 0.09 ^k^
Ningjing 48	6.17 ± 0.10 ^h^	25.92 ± 0.28 ^c^
Ningjing 43	6.53 ± 0.06 ^g^	11.93 ± 0.10 ^m^
Fuyuan 4	5.63 ± 0.04 ^i^	21.59 ± 0.06 ^e^
Songjing 22	3.62 ± 0.04 ^lm^	11.47 ± 0.11 ^n^
Songjing 19	5.67 ± 0.08 ^i^	18.73 ± 0.17 ^h^
Wuyoudao 4	4.70 ± 0.09 ^j^	7.32 ± 0.14 ^q^
Suijing 18	3.26 ± 0.08 ^n^	28.63 ± 0.08 ^b^
Suijing 309	2.75 ± 0.11 ^p^	29.68 ± 0.12 ^a^
Jiudao 75	11.61 ± 0.14 ^d^	20.83 ± 0.29 ^f^
Liaojing 337	4.63 ± 0.11 ^j^	13.98 ± 0.24 ^kl^
Yujing 91	4.40 ± 0.07 ^k^	9.07 ± 0.17 ^p^
Jingliangyouhuazhan	4.30 ± 0.04 ^k^	8.83 ± 0.07 ^p^
Tianyouhuazhan	3.39 ± 0.09 ^n^	10.67 ± 0.16 ^o^
Xinjing 64	16.97 ± 0.15 ^a^	24.06 ± 0.17 ^d^
Zhennuo 19	16.91 ± 0.13 ^a^	17.31 ± 0.23 ^d^
Longdao 24	13.17 ± 0.11 ^b^	13.87 ± 0.26 ^d^
Longjing 31	11.98 ± 0.09 ^c^	12.50 ± 0.14 ^l^

Note: Within a column, values followed by different superscript letters are significantly different at *p* < 0.05, according to Duncan’s multiple range test.

**Table 3:** Effect of water-to-rice ratio on slurry texture.

Ratio	Texture Description
1:0.5	Extremely viscous, non-stirrable
1:1	Highly viscous, difficult to stir
1:2	Moderately viscous, stirrable
1:4	Slightly thin
1:6	Very thin
1:8	Extremely thin

**Table 4:** Optimized parameters for GABA-enriched beverage production.

Processing Stage	Optimized Condition
Raw material	Suijing 309 brown rice
Germination	4 h soaking (RT) + 40 h sprouting (30 °C, rinsing every 2 h)
Slurry preparation	Rice-to-water ratio 1:2 (*w*/*v*)
Liquefaction	α-amylase 30 U/g, 80 °C, 40 min
Saccharification	Glucoamylase 75 U/g, 60 °C, 1 h
Fermentation consortium	10-strain Culture J (*Lactobacillus* and *Bifidobacterium* consortium)
MSG supplementation	0.25% (*w*/*v*)
Inoculum size	4% (*w*/*v*)
Cofactor addition	Pyridoxine hydrochloride 10 μmol/L
Fermentation	37 °C, 24 h

**Table 5:** Nutritional profile of the fermented beverage.

Parameter	Value
Energy	233.58 kJ/100 g
Protein	0.057 g/100 g
Fat	0.2 g/100 g
Carbohydrates	13.45 g/100 g
Sodium	112.2 mg/100 g
Lactic acid bacteria	2 × 10^8^ CFU/g
Total dietary fiber	0.21 g/100 g
Reducing sugars	14.6 g/100 g
GABA	12.2 mg/100 g

## Data Availability

The original contributions presented in the study are included in the article/Supplementary Material. Further inquiries can be directed to the corresponding author.

## Supplementary Materials

The following supporting information can be downloaded at: https://www.mdpi.com/article/10.3390/foods14152733/s1, Table S1. Rice Varieties, Origins, and Types.
