# Supplementary material for: Dual Bioconversion Strategy: Synergistic Germination and Lactobacillus Fermentation Engineering for a γ-Aminobutyric Acid-Enriched Beverage from Brown Rice
Source: Foods. 2025 Aug 5;14(15):2733. doi: 10.3390/foods14152733 (PMC12345850; doi:10.3390/foods14152733)
Supplement: Supplementary file 1 [file foods-14-02733-s001.zip › foods-3771532-supplementary.pdf]

Supplementary material

Dual Bioconversion Strategy: Synergistic Germination and *Lactobacillus* Fermentation Engineering for γ-Aminobutyric Acid-Enriched Beverage from Brown Rice

Di Yuan[1,2,3], Shan Zhang[1],Bin Hong[1],Shan Shan[1], Jingyi Zhang[1], Dixin Sha[1], Shiwei Gao[4], Qing Liu[4], Shuwen Lu[1,*] and Chuanying Ren[1,*]

1 Food Processing Research Institute, Heilongjiang Academy of Agricultural Sciences, Harbin 150086, China; yuandi199707@163.com (D.Y.); zhangshanfood@163.com (S.Z.); gru.hb@163.com (B.H.); 18845896856@163.com (S.S.); 18846080235@139.com (J.Z.); shadixin1997@163.com (D.S.);

2 Heilongjiang Province Key Laboratory of Food Processing, Harbin 150086, China

3 Heilongjiang Province Engineering Research Center of Whole Grain Nutritious Food, Harbin 150086, China

4 Suihua Branch of Heilongjiang Academy of Agricultural Sciences, Suihua 152001, China; gaoshiwei1118@126.com (S.G.); liuqing58627@163.com (Q.L.)

* Correspondence: shuwenl@sina.com (S.L.); chuanying1023@163.com (C.R.)

Table S1. Rice Varieties, Origins, and Types.

**Sample Name**	Origin (Province)	Type
Zhongjiazao 17	Zhejiang	Indica
Yixiangyou 2115	Sichuan	Indica
Deyou 8	Yunnan	Indica
Fengliangyou 4	Hubei	Indica
Tiejing 11	Liaoning	Japonica
Tonghe 899	Jilin	Japonica
Tongke 59	Jilin	Japonica
Ningjing 48	Ningxia	Japonica
Ningjing 43	Ningxia	Japonica
Fuyuan 4	Ningxia	Japonica
Songjing 22	Heilongjiang	Japonica
Songjing 19	Heilongjiang	Japonica
Wuyoudao 4	Heilongjiang	Japonica
Suijing 18	Heilongjiang	Japonica
Suijing 309	Heilongjiang	Japonica
Jiudao 75	Jilin	Japonica
Liaojing 337	Liaoning	Japonica
Yujing 91	Hunan	Indica
Jingliangyouhuazhan	Hunan	Indica
Tianyouhuazhan	Hunan	Indica
Xinjing 64	Henan	Japonica
Zhennuo 19	Anhui	Glutinous
Longdao 24	Heilongjiang	Japonica
Longjing 31	Heilongjiang	Japonica
